# Association of urinary citrate excretion, pH, and net gastrointestinal alkali absorption with diet, diuretic use, and blood glucose concentration

**DOI:** 10.14814/phy2.13411

**Published:** 2017-10-16

**Authors:** Majuran Perinpam, Erin B. Ware, Jennifer A. Smith, Stephen T. Turner, Sharon L. R. Kardia, John C. Lieske

**Affiliations:** ^1^ Division of Nephrology and Hypertension Mayo Clinic Rochester Minnesota; ^2^ Institute for Social Research University of Michigan Ann Arbor Michigan; ^3^ Department of Epidemiology School of Public Health University of Michigan Ann Arbor Minnesota; ^4^ Department of Laboratory Medicine and Pathology Mayo Clinic Rochester Minnesota

**Keywords:** Citrate, diabetes mellitus, diet, glucose, nephrolithiasis, pH

## Abstract

Urinary citrate (Ucit) protects against urinary stone formation. Acid base status and diet influence Ucit. However, the effect of demographics, diet, and glucose metabolism on Ucit excretion, urinary pH (U‐pH) and net gastrointestinal alkali absorption (NAA) are not known. Twenty‐four hour urine samples, blood glucose, creatinine, and cystatin C were obtained from non‐Hispanic white sibships in Rochester, MN (*n* = 446; 64.5 ± 9 years; 58% female). Diet was assessed by a food frequency questionnaire. The impact of blood glucose, demographics and dietary elements on Ucit excretion, U‐pH, and NAA were evaluated in bivariate and multivariable models and interaction models that included age, sex, and weight. NAA significantly associated with Ucit and U‐pH. In multivariate models Ucit increased with age, weight, eGFR_C__ys_, and blood glucose, but decreased with loop diuretic and thiazide use. U‐pH decreased with serum creatinine, blood glucose, and dietary protein but increased with dietary potassium. NAA was higher in males and increased with age, weight, eGFR_C__ys_ and dietary potassium. Significant interactions were observed for Ucit excretion with age and blood glucose, weight and eGFR_C__ys,_ and sex and thiazide use. Blood glucose had a significant and independent effect on U‐pH and also Ucit. This study provides the first evidence that blood glucose could influence urinary stone risk independent of urinary pH, potentially providing new insight into the association of obesity and urinary stone disease.

## Introduction

Kidney stones are common, affecting up to 10% of people during their lifetime (Scales et al. 2012). Citrate is thought to be an important crystallization inhibitor, and oral potassium citrate is often a first line of treatment for preventing stone recurrence (Pearle et al. 2014). Citrate complexes with filtered calcium, and also has independent effects at the crystal surface to inhibit calcium oxalate and brushite crystal growth (Ryall 1997; Pearle et al. 2014). Some filtered citrate is reabsorbed in the proximal tubule, largely regulated by proximal tubule cell pH (Zuckerman and Assimos 2009). Hypocitraturia is found in 20–60% of stone formers (Zuckerman and Assimos 2009). Treatment is of the underlying disorder as well as administration of potassium citrate which is thought efficacious for hypocitraturia caused by distal renal tubular acidosis (RTA), chronic diarrheal syndrome, and/or thiazide‐induced hypokalemia (Zuckerman and Assimos 2009).

Daily acid generation is greatly influenced by diet (Sakhaee et al. 1993), making it an important factor when evaluating a kidney stone patient. Urinary pH is also an important factor in stone risk, with higher values favoring calcium phosphate stones and lower ones uric acid stones (Pearle et al. 2014). Although final urine pH is associated with acid load, it is also influenced by ammonium excretion and the ability of the distal tubule to acidify the collecting duct. Overall factors that influence urine pH and citrate are similar, but not identical.

Stone formers with noninsulin dependent diabetes mellitus have a higher frequency of uric acid stones than nondiabetic stone formers (Sakhaee 2014), in part due to effects of insulin resistance on urinary ammonium levels (Li et al. 2014). However, the independent effect of blood glucose on urinary citrate excretion has not been determined. Furthermore, while urinary citrate and urine pH are important in kidney stone pathogenesis, very few studies have examined the effects of demographics on either (Hosking et al. 1985; Trinchieri et al. 1992). Also, studies to date have relatively small sample sizes, present conflicting evidence, or have not accounted for diet. In this study, we took advantage of detailed demographic, environmental, and urinary data in a well characterized cohort to examine the determinants of urinary pH, citrate excretion, and net gastrointestinal alkali absorption.

## Methods

This study was approved by the Mayo Clinic Institutional Review Board (Mayo IRB# 08‐006238” Genetic Determinants of Urine Lithogenicity ‐ Working Protocol”).

### GENOA cohort

The multi‐phase Genetic Epidemiology Network of Arteriopathy (GENOA), a member of the Family Blood Pressure Program (FBPP), recruited non‐Hispanic white hypertensive sibships from Rochester, Minnesota for linkage and association studies to investigate the genetic underpinnings of hypertension in Phase I (1996–2001) (Daniels et al. 2004). The Genetic Determinants of Urinary Lithogenicity (GDUL) study (2006–2012) is an ancillary study of the Phase III GENOA Genetics of Chronic Kidney Disease (CKD) study conducted in GENOA participants (Rule et al. 2013). Participants in the Rochester, MN GENOA cohort were invited to collect 24 h urine samples and complete a food frequency questionaire (FFQ, Viocare Technologies, Princeton, NJ) (Kristal et al. 2014). Participants were excluded from this study if they were in end‐stage renal failure (stage 5 CKD).

### Study visit

After informed consent, participants completed at least one 24 h urine collection (Hess et al. 1997; Parks et al. 2002) and the FFQ at a CKD and/or GDUL study visit. A total of 91 (20.4%), 202 (45.3%), and 153 (34.3%) participants had a total of one, two, or three urine collections, respectively. For individuals with two or three urine collections, values were averaged for analysis. The mean time between the earliest (CKD) and latest (GDUL) urine collections was 1.73 years (range = 0.9–3.6 years). The average time between the two GDUL collections was 22 days. Intraclass correlation coefficients (ICCs) for urine factors across collections revealed that the majority of urine measures were relatively stable across time. The ICC for pH was 0.50, for net alkali absorption was 0.39, and for citrate was 0.65. Participants also completed a detailed kidney stone questionnaire (to assess stone‐forming status and other medical comorbidities and risk factors), and data from a GENOA CKD Study Questionnaire was available as needed.

### Urine collection

Urine was collected with toluene as a preservative. Twenty‐four hour urinary concentration of citrate, urine pH and other determinants of supersaturation were measured in the Mayo Clinic Renal Testing Laboratory. Serum creatinine was assessed using a standardized enzymatic assay on a Roche Cobas chemistry analyzer (c311) (Roche Diagnostics; Indianapolis) while cystatin C was measured using an immunoturbidometric assay (Gentian; Moss, Norway) that was traceable to an international reference material. Glomerular filtration rate (GFR) was independently estimated using cystatin C (eGFR_Cys_) (Inker et al. 2012).

### Descriptive statistics

Data management and statistical analyses were conducted in SAS version 9.3 (SAS Institute Inc., Cary) (R Development Core Team, 2008). Urine measures appeared to have relatively normal distributions; thus, no variable transformations were applied. Values that were ≥4 standard deviations from the mean of any urine or diet measure were removed. Linear mixed effects models (LMM), that included sibship as a random intercept (to properly account for family structure), were used to test whether there were significant differences by sex for the urinary and diet measures.

### Variable selection

A randomly selected, independent subset of the GENOA cohort (one individual per sibship) was used for stepwise linear regression to determine the variables that were associated with urinary citrate, pH and net alkali absorption. Variables available for selection included: weight, body mass index (BMI), smoking status (current‐ or never smoker), diabetes status (yes/no), recent fasting blood glucose level (within 1 year of the urine collection), systolic blood pressure (SBP), diastolic blood pressure (DBP), eGFR_Cys_, diuretic loop use (yes/no), diuretic thiazide use (yes/no), and dietary animal protein, sodium, calcium, fructose, oxalate, total protein, and sucrose intakes. Net alkali absorption was calculated using the Oh method: (Urine(Na + K  +  Ca + Mg)‐(Cl + 1.8P))(Oh 1989). The entry criterion for stepwise selection was *P *< 0.05, and the exit criterion was *P *> 0.10. Variables that are known to strongly influence urinary citrate (age, sex, serum creatinine, and dietary potassium) were forced into each model.

### Relationships between urinary chemistries

**Figure 1:**
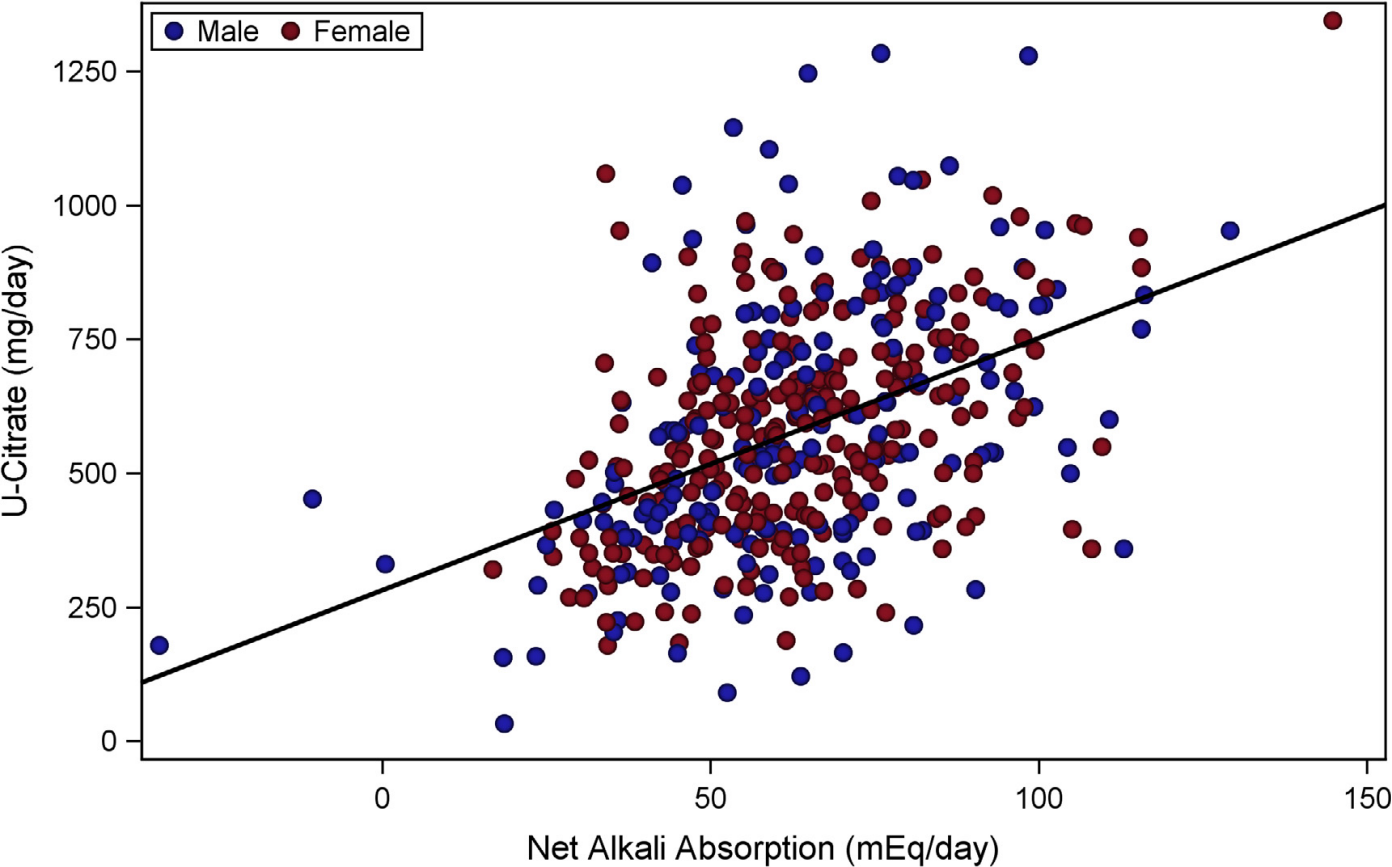
Urinary citrate versus net alkali absorption. Adjusted urinary citrate was significantly, positively associated with adjusted net alkali absorption (*β* = 4.7062; *P *< 0.0001). Urinary citrate was adjusted for age, sex, serum creatinine, dietary potassium intake, weight, blood glucose, eGFR_cys_, diuretic loop use, and diuretic Thiazide use. Net alkali absorption was adjusted for age, sex, serum creatinine, dietary potassium, eGFR_cys_, and diuretic loop use.

**Figure 2:**
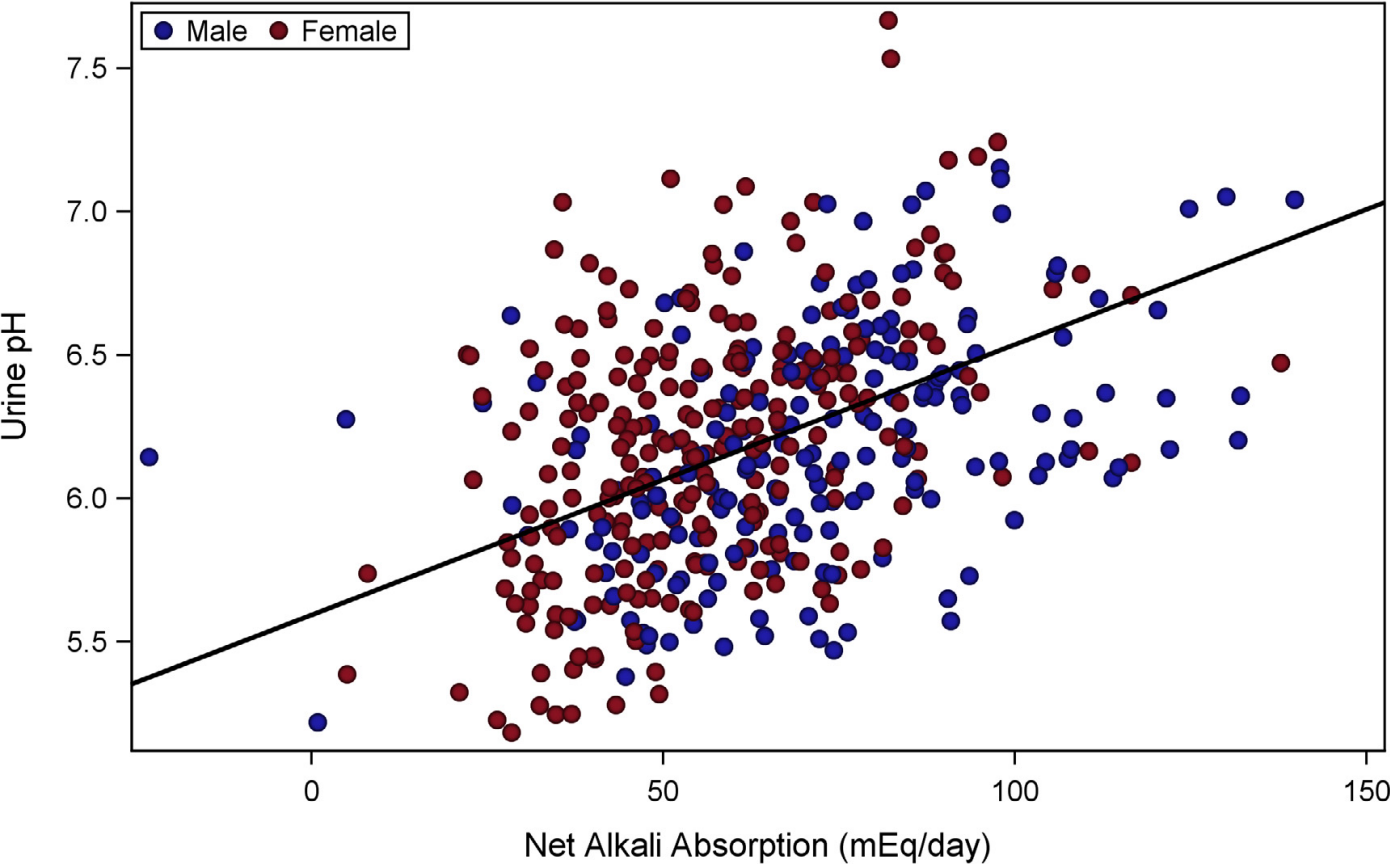
Urine pH versus net alkali absorption. Adjusted urine pH was significantly, positively associated with adjusted net alkali absorption values (*β* = 0.009; *P *< 0.0001). Urine pH was adjusted for age, sex, serum creatinine dietary potassium intake, blood glucose, diuretic loop use, and dietary protein intake. Net alkali absorption was adjusted for age, sex, serum creatinine, dietary potassium, eGFR_cys_, and diuretic loop use.

**Figure 3:**
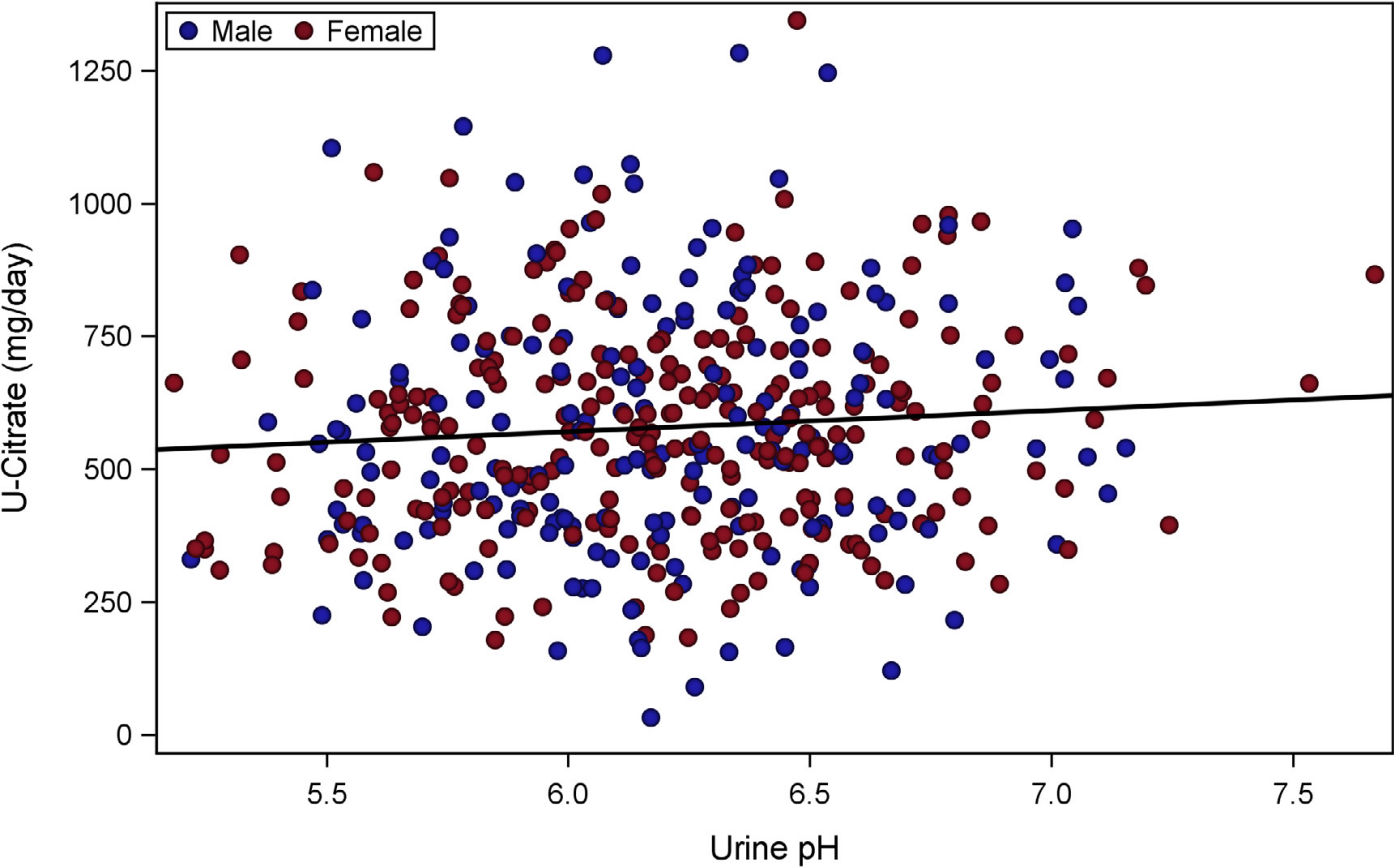
Urinary citrate versus urine pH. Adjusted urinary citrate was not significantly associated with urine pH (*β* = 40.18; *P* = 0.10). Urinary citrate was adjusted for age, sex, serum creatinine, dietary potassium intake, weight, blood glucose, eGFR_cys_, diuretic loop use, and diuretic Thiazide use. Urine pH was adjusted for age, sex, serum creatinine dietary potassium intake, blood glucose, diuretic loop use, and dietary protein intake.

To evaluate and visualize the relationship between citrate and net alkali absorption (Fig. 1), pH and net alkali absorption (Fig. 2), and pH and citrate (Fig. 3), a two‐stage modeling approach was used. In stage one, the urine chemistries of interest were first adjusted for variables from the stepwise selection process, separately. The mean value of the urine chemistry variable was then added back to the raw residuals, and the resulting adjusted residuals were used as either the exposure or outcome variables for stage two. The stage two model included the adjusted residuals of citrate (Figs. 1 and 3) or pH (Fig. 2) from stage one as the dependent variable, and the adjusted residuals of net alkali absorption (Figs. 1 and 2) or pH (Fig. 3) from stage one as the independent variable. The dependent and independent variables were then plotted in a scatter plot, and linear regression lines were overlaid.

### Association testing

After variable selection, LMM was performed on the GENOA subset sample with complete data on all selected variables (*n* = 446) to assess significant predictors of the urinary measures, accounting for the sibship structure in GENOA. Interaction models were also conducted to assess age, sex, and weight (if weight was included in the model selection as a predictor) interactions with the variables included in the models. Interactions were considered significant at an alpha level of 0.05. Sensitivity analyses were performed by running the final multivariable models in the subset of participants with no history of kidney stones to assess direction and significance of the final variables.

### Interaction plots

**Figure 4:**
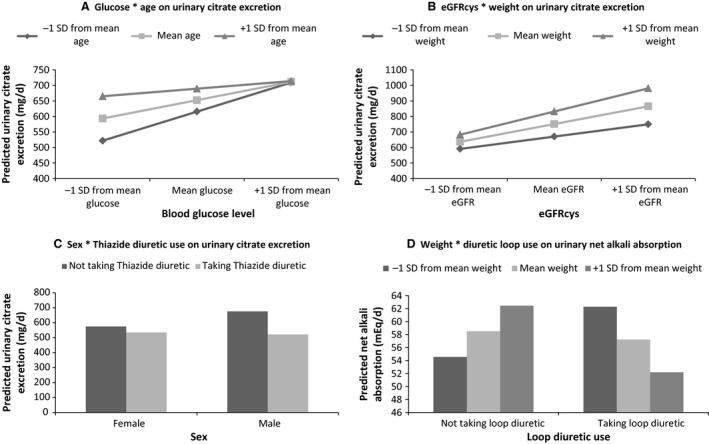
Interaction models. (A) Age and blood glucose on urinary citrate excretion. Higher age decreased the effect of blood glucose on urinary citrate excretion (*β*_age x blood glucose_ = −0.20, *P* = 0.01). SD, age (years), blood glucose (mg/dL). (B) eGFR_C__ys_ and weight on urinary citrate excretion. Higher weight increased the effect of eGFR_C__ys_ on urinary citrate excretion (*β*_weight x_ _eGFR__cys_ = 0.07, *P* = 0.01). eGFRcys: estimated Glomular Filtration Rate (mL/min/1.73 m^2^), weight in kg, SD. (C) Sex and Thiazide diuretic use on urinary citrate excretion. Thiazide use decreased urinary citrate excretion more in males than females (*β*_sex x thiazide use_ = −114.22, *P* = 0.04). SD. (D) Weight and loop diuretic use on net alkali absorption. Individuals taking loop diuretics showed increased net alkali absorption as weight increased, while those not taking loop diuretics showed a decreased net alkali absorption as weight increased. (*β*_weight x loop diuretic use_ = −0.45, *P* = 0.04).

Fig. 4A–D were created to visualize the blood glucose x age (4A); eGFR_cys_ x weight (4B); and sex x thiazide diuretic use (4C) interactions on urinary citrate excretion, as well as the interaction between loop diuretic use and weight on net alkali absorption (4D). Multivariable regression models that included the stepwise selection variables were run for each interaction. To calculate predicted urinary outcome, the beta estimates for continuous predictor variables were multiplied by the mean value for the respective variable from the sample, and for dichotomous variables, the reference group was used where appropriate (i.e., female, not taking diuretics). Three values for the continuous variables of interest (age and blood glucose (Fig. 4A); eGFR_cys_ and weight (Fig. 4B and D) were obtained by taking the mean value of each of the variables, and the mean value adding or subtracting one standard deviation. These values were used to produce nine predicted urinary citrate values (at each of the three values for the two variables of interest) that were plotted in the interaction plots for blood glucose x age (Fig. 4A) and eGFR_cys_ x weight (Fig. 4B) and six predicted net alkali absorption values for the weight x loop diuretic use interaction (Fig. 4D). For the sex x thiazide diuretic use plot (Fig. 4C), four predicted urinary citrate excretions were calculated to create the interaction plot (females taking diuretics, males taking diuretics, females not taking diuretics, males not taking diuretics).

## Results

### Descriptive statistics

A total of 446 individuals from 303 sibships participated in this study (Table 1). The sibship structure of the sample was as follows: 198 singletons, 82 sibpairs, 15 sibships with 3 siblings and 8 sibships with 4 or more siblings. The mean age was 64.5 ± 9 years and 58% of the participants were female. Three individuals took potassium citrate medication. Out of 446 participating individuals, 408 provided information on kidney stone history, of whom 47 (11.5%) had reported a history of stones, consistent with stone prevalence in the general population (Pearle et al. 2014). Sensitivity analyses performed in the subset of nonstone formers showed consistent results (direction and significance) for each of the multivariable models.

**Table 1:** Descriptive statistics

	Combined *n* = 446	Female *n* = 259	Male *n* = 187	*P*‐value
	Mean (SD) or *n* (%)	Mean (SD) or *n* (%)	Mean (SD) or *n* (%)	
Age, year	64.5 (9.0)	63.4 (9.1)	66.1 (8.7)	0.0077
Weight, kg	88.7 (19.8)	81.8 (18)	98.2 (18.3)	<0.0001
BMI, kg/m^2^	31.0 (6.0)	30.7 (6.5)	31.4 (5.2)	0.222
Serum creatinine, mg/dL	0.8 (0.2)	0.8 (0.2)	1.0 (0.2)	<.0001
eGFR_Cys_, mL/min/1.73 m^2^	86.8 (24.6)	90.0 (25.5)	82.3 (22.6)	0.0081
History of stone formation^a^	47 (11.5)	21 (8.8)	26 (15.3)	0.0482
Diabetes status				0.0125
Yes	55 (12.3)	23 (8.9)	32 (17.1)	
No	391 (87.7)	236 (91.1)	155 (82.9)	
Blood glucose, mg/dL	95.2 (18.9)	92.8 (19.1)	98.6 (18.1)	0.012
Dietary measures				
Total protein, g/day	80.6 (33.9)	75.2 (29.6)	88.1 (38.0)	<.0001
Potassium, mg/day	3164.7 (1269.8)	3138.1 (1255.7)	3201.6 (1291.6)	0.5941
*Diuretic use*				
Loop				0.2321
Yes	18 (4.0)	13 (5.0)	5 (2.7)	
No	428 (96.0)	246 (95.0)	182 (97.3)	
Thiazide				0.9228
Yes	166 (37.2)	96 (37.1)	70 (37.4)	
No	280 (62.8)	163 (62.9)	117 (62.6)	
Urinary traits				
Citrate, mg/day	578.9 (320.1)	542.2 (284.7)	629.6 (358.2)	0.0017
pH	6.2 (0.5)	6.3 (0.5)	6.1 (0.5)	0.0004
Net alkali absorption, mEq/day	71.0 (25.2)	64.5 (21.6)	80.0 (26.9)	<0.0001

*P*‐values were testing for sex differences, using linear mixed models to account for sibships.

SD, standard deviation, BMI, body mass index, eGFR, estimated Glomerular Filtration Rate (cystatin calculation).

a Total sample size for history of stone formation is 408.

### Relationships between urinary chemistries

Urinary citrate, adjusted for age, sex, serum creatinine, dietary potassium intake, weight, blood glucose, eGFR_cys_, and diuretic use, was significantly and positively associated with net gastrointestinal alkali absorption, adjusted for age, sex, weight, serum creatinine, dietary potassium intake, eGFR_cys_, and loop diuretic use (*β* = 4.70; *P *< 0.0001). Urine pH adjusted for age, sex, serum creatinine dietary potassium intake, blood glucose, loop diuretic use, and dietary protein intake also positively associated with adjusted net gastrointestinal alkali absorption (*β* = 0.009; *P *< 0.0001). However, adjusted citrate and adjusted urine pH were only weakly and not significantly associated (*β* = 40.18; *P* = 0.10).

### Association testing

In the bivariate analysis (Table 2) there was an age‐related decline in urinary citrate. Males had significantly higher urinary citrate, higher net alkali absorption and lower pH than females. Urinary citrate was positively associated with weight, eGFR_Cys_, and blood glucose, and negatively associated with serum creatinine and loop diuretic use. Urinary pH decreased with increasing serum creatinine and blood glucose, and was also decreased for those taking loop diuretics.

**Table 2:** Bivariate associations for urine citrate, pH, and net alkali absorption

	Urine citrate, mg/day	Urine pH	Net alkali absorption
	*β*	*β*	mEq/day, *β*
Age, year	−3.725^a^	−0.003	0.280^a^
Sex (male)	96.284^b^	−0.173^c^	14.819^c^
Weight, kg	3.480^c^		0.191^c^
Serum creatinine, mg/dL	−222.420^b^	−0.633^c^	6.343
eGFR_Cys_, mL/min/1.73 m^2^	3.653^c^		0.074
Blood glucose, mg/dL	3.917^c^	−0.007^c^	
*Dietary measures*			
Total protein, g/day			
Potassium, mg/day	0.023	0.00003	0.003^c^
Diuretic use			
Loop (yes)	−289.620^c^	−0.315^a^	−12.640^a^
Thiazide (yes)	−65.139^a^	−0.001	

*β*, beta estimate, eGFR, estimated Glomerular Filtration Rate (cystatin calculation);

a *P* < 0.05.

b *P* < 0.01.

c *P* < 0.001.

In the multivariable model (Table 3) urinary citrate increased with age, weight, eGFR_Cys_, and blood glucose, and decreased with loop diuretic and thiazide use. Urine pH decreased with serum creatinine, blood glucose and dietary protein and increased with dietary potassium. Net alkali absorption was higher in males and increased with age, weight, eGFR_Cys_ and dietary potassium.

**Table 3:** Multivariable associations for urine citrate, pH, and net alkali absorption

	Urine citrate, mg/day	Urine pH	Net alkali absorption
	*β*	*β*	mEq/day, *β*
Intercept	−670.95^b^	7.25^c^	−21.15
Age, year	4.41^a^	−0.0008	0.58^c^
Sex (male)	58.26	0.01	12.26^c^
Weight, kg	3.94^c^		0.16^b^
Serum creatinine, mg/dL	−144.39	−0.5^c^	−2.29
eGFR_Cys_, mL/min/1.73 m^2^	4.19^c^		0.21^c^
Blood glucose, mg/dL	3.56^c^	−0.01^c^	
Dietary measures			
Total protein, g/day		−0.0045^c^	
Potassium, mg/day	0.02	0.0001^c^	0.0033^c^
Diuretic use			
Loop (yes)	−238.36^c^	−0.21	−7.15
Thiazide (yes)	−87.74^b^		

*β*, beta estimate, eGFR, estimated Glomerular Filtration Rate (cystatin calculation).

a *P* < 0.05.

b *P* < 0.01.

c *P* < 0.001.

Three significant interactions were observed (Fig. 4A–C) for urinary citrate excretion including age and blood glucose (*β* = −0.20, *P* = 0.01), weight and eGFR_Cys_ (*β* = 0.07, *P* = 0.01), and sex and thiazide use (*β* = −114.22, *P* = 0.04). One significant interaction for net alkali absorption with weight and loop diuretic use was also observed (Fig. 4D; *β* = −0.45, *P* = 0.04).

## Discussion

In this study, we assessed the effects of demographics, metabolic parameters, and diet on urinary citrate, pH, and net alkali absorption. As previously described, net gastrointestinal alkali absorption influenced urinary citrate (Sakhaee et al. 1993), as well as urinary pH. We also found that a higher fasting glucose was associated with a lower urinary pH, but a higher urinary citrate. This observation may shed new insights into the association of obesity and diabetes with urinary stone risk, especially for uric acid stones.

After adjusting for covariates, urinary citrate increased with age, consistent with observations from a recent study restricted to stone formers alone (Friedlander et al. 2014). Hosking and colleagues also observed an increased citrate excretion with age among healthy individuals (*n* = 83), but not stone formers (*n* = 132) (Hosking et al. 1985). Another study in nonstone formers found that the daily urinary excretion of citrate increased with age, to reach a maximum during the fifth decade, then remaining relatively constant thereafter until the eight decade when it decreased (*n* = 197). However, only 14 individuals were >60 years old (Trinchieri et al. 1992). Thus, to more accurately assess age‐related effects on urinary citrate excretion, it may be important to distinguish between stone formers and nonstone formers, since hypocitraturia is found in 20–60% of persons with urinary stone disease for reasons yet to be ascertained (Zuckerman and Assimos 2009).

Kidney function together with renal ammoniagenesis decreases with age resulting in more acidic urine, since under these circumstances protons are disproportionately excreted as titratable acids. Thus, perhaps not surprisingly, urine pH also decreased with age in our study (Table 2). However, the net effect of these changes on systemic acid base balance is not clear, and the relationship between urine pH, ammonium excretion, citrate excretion, and normal aging is not necessarily predictable (Zuckerman and Assimos 2009). Our finding of an association between eGFR_Cys_ and urinary citrate excretion suggests the net effect is to reduce urine citrate (Table 3), perhaps as part of the compensation for reduced urinary ammonium excretion, and Gershman and colleagues also reported that reductions in GFR are associated with decreased urine citrate (Gershman et al. 2012).

Most importantly, we demonstrate for the first time that having a higher fasting glucose is associated with higher urinary citrate, but lower urine pH (Table 3). Although a study by Pigna et al. (2014) did not find the correlation between urinary citrate excretion and blood glucose significant (*P* = 0.12), likely due to a low number of participants (*n* = 21), the direction of effect was the same as that in the current study. Similar findings were also reported in a study of 252 stone formers (Daudon et al. 2006) in which diabetic uric acid and calcium stone formers had higher blood glucose and increased urinary citrate compared to nondiabetic stone formers. Among calcium stone formers urine pH was also significantly lower in the diabetic group compared with nondiabetics. Nevertheless, in this study blood glucose, but not diabetes status was an independent predictor of urine citrate. It is possible that frank diabetes has different effects on urine citrate, or that treatments for diabetes may confound the association. However, some of the effects of higher blood glucose are likely related to insulin which stimulates glycolysis (Saltiel and Kahn 2001). The increased citrate levels could stimulate acetyl‐CoA carboxylase and fatty acid synthesis (Martin and Vagelos 1962). Intracellular citrate can eventually be metabolized to bicarbonate, with the net effect being inhibition of citrate reabsorption in the proximal tubule (Simpson 1967; Moseley et al. 2013). However, insulin also alters adenosine triphosphate citrate lyase activity which converts citrate and CoA to oxaloacetate and acetyl‐CoA (Zuckerman and Assimos 2009; MacDonald et al. 2013). Overall, little is known about the net effects of blood glucose and insulin on urinary citrate excretion, and further studies are needed to elucidate underlying mechanisms. Furthermore, medications that alter insulin sensitivity could also affect urinary citrate and stone risk.

Mandel and colleagues performed a cross‐sectional study regarding urinary citrate on 2561 individuals from three large cohorts (Health Professionals Follow‐up Study (HPFS), Nurses’ Health Studies (NHS I and II) and found diabetes to be associated with higher urinary citrate excretion in men and younger women (Mandel et al. 2013). It was hypothesized that citrate could be competing with glucose for proximal tubule reabsorption through the sodium cotransport. The majority of citrate reabsorption in the kidney is thought to occur through NaDC1, a sodium‐dependent dicarboxylate co‐transporter in the proximal tubule (also expressed in small intestinal cells) (Pajor 1995; Ho et al. 2007). Since NaDC1 is also responsible for reabsorption of other TCA cycle intermediates, it could indeed be related to citrate reabsorption and glucose metabolism. Furthermore, our interaction model also revealed that the effect of blood glucose on urinary citrate excretion decreased with age (Fig. 4A), raising questions regarding age‐related changes in the relation between citrate reabsorption and glucose. While the effect of age on urinary citrate excretion was relatively small in this study (*β* = 4.41), this relationship provides important insights, since the relation between age and urinary citrate interacted significantly with blood glucose (Fig. 4A), suggesting glucose as an important factor. Indeed having higher blood glucose diminished the effect of age on urinary citrate.

Higher body weight was also associated with greater urinary citrate excretion (Table 3). Powell and colleagues studied 5942 stone formers of which 414 were obese (>120 kg in males >100 kg in females) and also found the obese group to have a higher 24‐h urinary citrate excretion (Powell et al. 2000). Conversely, Mandel and colleagues found having high BMI was associated with a lower urinary citrate (Mandel et al. 2013). Since we found an effect of weight, but not BMI on urinary citrate in the main effects model, it suggests that body habitus is important. Citrate metabolism is known to be affected by fatty acid synthesis (Martin and Vagelos 1962) and thus could explain the increased citrate levels with higher weight. Individuals with greater weight were also found to have a steeper increase in urinary citrate levels at higher levels of eGFR_Cys_ (Fig. 4B). This could be related to the body weight itself and/or altered food intake mix related to greater body weight. However, body habitus could be more important than diet. A study in mice found NaDC1−^/^− animals demonstrated no significant alteration in serum citrate levels, suggesting that metabolic citrate production is more important than citrate absorption via the gut (Ho et al. 2007).

A lower urinary pH has also been observed in those with a higher BMI (Maalouf et al. 2004) and higher fasting glucose (Otsuki et al. 2011). Insulin resistance and/or accumulation of lipids in renal proximal tubular cells leading to reduced renal ammoniagenesis have been implicated as factors contributing to these observations (Maalouf et al. 2010). These mechanisms likely contribute to the lower urinary pH we observed with higher fasting glucose.

We found thiazide use associated with decreased urinary citrate, as previously shown (Mandel et al. 2013). This is likely caused by thiazide‐induced hypokalemia, which would stimulate citrate reabsorption in the proximal tubules (Zuckerman and Assimos 2009). Loop diuretic use also decreased urinary citrate (Table 3), probably also due to hypokalemia. Thiazides were found to reduce urinary citrate excretion more in males than females (Fig. 4C), suggesting males taking thiazides might potentially require closer monitoring for this complication.

Several interesting associations with urinary citrate excretion were observed, some of which paralleled effects on urine pH and others which did not. Urine pH decreased with protein intake which can be explained by the increased acid load from protein. Dietary potassium increased urine pH, perhaps due to relatively higher ingestion of fruits and vegetables (Pearle et al. 2014).

Our study has limitations. Participants were of European descent and of relatively older age. Eisner and colleagues found Asian/Pacific islander stone formers to have less urinary citrate excretion than Whites (Eisner et al. 2010). Our population was also largely nonstone forming, however stone formers are likely to have altered acid‐base balance (Zuckerman and Assimos 2009) and intentional diet changes. Also a fasting glucose would ideally be obtained at the exact time of the urine collection. However, fasting glucose is not likely to change significantly over a short period of time unless diabetes status changes (e.g., with weight loss or gain). However, it is also important to recognize that the observed associations of fasting blood glucose with urinary parameters apply at a population level and point toward a physiologic mechanism, but cannot be used to predict urinary parameters on an individual level due to within person variability (Sacks 2011). Despite these limitations, the current study benefits from a large sample size and inclusion of dietary data. Studying healthy individuals allowed us to examine the actual influence of age, sex and other parameters on urinary citrate excretion, and how changes in urinary citrate excretion can explain trends in kidney stone events.

In conclusion, our study points toward the ambient blood glucose concentration as having a measurable unique influence on urinary citrate excretion and pH. While age and weight also associated with urinary citrate excretion, the effect of age interacted with blood glucose levels, rather than reduced kidney function. A deeper understanding of the relation between blood glucose and urinary citrate excretion could increase our understanding of the interrelationship between obesity, insulin resistance, urinary citrate, and kidney stone risk.

## Conflict of Interest

The authors declare that they have no conflict of interest.
